# Ultra-Wide Band Double-Slot Podal and Antipodal Vivaldi Antennas Feed by Compact Out-Of-Phase Power Divider Slot for Fluid Properties Determination

**DOI:** 10.3390/s22124543

**Published:** 2022-06-16

**Authors:** Jiwan Ghimire, Dong-You Choi

**Affiliations:** Department of Information and Communication Engineering, Chosun University, Gwangju 61452, Korea; ghimire@chosun.kr

**Keywords:** UWB, vivaldi antenna, podal, antipodal, viscosity, radar, microstrip power divider, polar fluids

## Abstract

In this paper, double slot podal and antipodal ultra-wideband (UWB) microstrip antennas for a fluid property measurement system are proposed. Among different feeding techniques, out of phase uni-planner power divider approach is used. The performance verification of the proposed antenna is explained, along with a performance comparison of the antenna bandwidth, feeding, and the realized gain. The suggested podal antenna has an impedance bandwidth from 2.4 to 15.4 GHz, with a maximum gain of 11.3 dBi in the 12 GHz region while the antipodal antenna has a 2.8 GHz to 16 GHz impedance bandwidth, with a maximum gain of 10.4 dBi in the 10 GHz region. Within the intended band, the radiation pattern had an excellent directivity characteristic. The implementation of the proposed antenna is calibrated by measuring the propagated signals response via various liquid specimens using UWB radar, which might be applied for fluid sensing and prediction purposes. The proposed antenna was connected to an NVA-R661 module of Xethru Inc. for measuring the sample delay and peak-to-peak amplitude of the received signals passing through specimens. The measured parameters at a different radar frequency range of transmission are applied by drawing the fluid viscous analogy based on Poiseuille’s law hypothesis, showing clear differentiation between the test specimens.

## 1. Introduction

Due to certain advantages such as low profile, compactness, cheap costs in system development, planar configuration, and ease of integration [1,2,3,4] over conventional measurement techniques such as using waveguide sample cells [5,6], resonant cavities or coaxial probes [7,8,9], magnetic material [10,11], horn antennas [12], a microstrip antenna have been considered as potential alternatives for detecting and monitoring the physical property and permittivity analysis of the material. Similarly, microstrip antennas have also been studied as a potential option for landmine detection [13], through-wall imaging [14], biomedical imaging [15], breathing, heart rate detection [16], motion and gesture detection [17], and two-dimensional (2D) imaging of concrete blocks [18]. Depending on the physical and dielectrics properties of the material, microwave sensing techniques have a wide range of industrial applications, such as identifying or monitoring material permittivity, density distribution, temperature, moisture content, and compositional proportions in materials [18,19,20,21,22,23,24]. The microstrip patch antenna is used to record the moisture level contained in rubber latex by observing the shift in the resonant frequency [25], as a sensor dipped into a liquid chamber for salinity determination [26], as a new method and the prototype system for measuring permittivity and properties of dielectric material by sensing the change of the input impedance of a transmitting microstrip patch antenna [27], as a temperature sensor with reactive impedance surface ground plane [28], as a method of detecting the dielectric characteristics of water based on the proportion of salt and sugar incorporated [29,30]. The process or techniques developed for detection and sensing require are complex and time-consuming, and the cost of developing these systems is high. Limitations in bandwidth at microwave frequency, materials, feeding space, installation, and application act as a constraint when analyzing using existing methods. As a result, the challenge of developing a method of measuring the constitutional properties of materials that is simple to use, least reliant on various parameters, and provides enough measurement accuracy remains crucial. To achieve the required penetration depth, especially through the high lossy test samples or specimens, an antenna with a wide frequency range, consistent unidirectional end-fire radiation character, and a high directed gain is preferred for microwave transmission. Different antenna designs are implemented for dielectric measurement systems such as crescent-shaped patch and slotted partial ground patch antenna [3,30], conventional microstrip patch antenna [21,26], EBG Based Microstrip Patch Antenna [15], microstrip rectangular patch with a grid pattern ground plane [28], SRR and CSRR-based microstrip sensor [31,32], and TEM horn antenna [33,34,35], depending on whether the application demands high gain, efficiency, and a consistent radiation pattern for improved sensitivity in the depiction of materials property. Several methods are proposed for achieving higher gain such as using dielectric [36] and metamaterial lenses [37], placing parasitic elliptical patch [38], electromagnetic bandgap (EBG) [39], adding profiled dielectric directors [40], applying negative index material (NIM) [41]. zero-index material (ZIM) [42], frequency-selective surface (FSS) [43], and dielectric slab [44]. However, achieving effective gain improvements using several known approaches in a small region within the antenna is still a complex, challenging, and time-consuming process. The feeding network determines the performance of an antenna. Because the feeding network part takes up most of the precious area, many of them are not symmetrically distributed. Therefore, the size of the feeding structures should be counted while constructing antennas. Various feeding array structures (e.g., SIW binary splitter, SIW power dividers, grounded coplanar waveguide (GCPW), T-junction power divider, combined T-type and Y-type dividers, four-way SIW power divider, a two-way power divider, and a 1-to-8 power divider network [45,46,47,48,49,50,51,52,53]) are proposed in various studies for improving overall antenna performance either in terms of optimizing insertion loss or increasing frequency bandwidth. However, most power dividers are in-phase power dividers designed to feed a certain number of antenna arrays with the same amplitude and phase at the output, which, when used in the system, results in beam splitting at a higher frequency. To overcome this limitation among different feeding techniques, uniform amplitude out of phase uniplanar power divider feeding approach over a large frequency range is used. The feeding is a T-junction formed by a slot line and a microstrip line for both podal and antipodal antenna designs. In the case of podal antennas, the microstrip line has a Chebyshev transformation layout with each end terminating as a two-feeding slot of Vivaldi antennas. This feeding arrangement results in a more uniform, highly directive constructive field distribution at the antenna’s radiating end and offers a wide antenna bandwidth.

In this work, for the first time, podal and antipodal microstrip Vivaldi antennas connected with UWB radar module (NVA-R661 of Xethru Co., Oslo, Norway) [54] as a sensor for polar fluid properties measurement are proposed. This arrangement limits the use of complex equipment like network analyzer. The radar module can be used as a low-cost alternative for the Vector Network Analyzer (VNA). The Xethru radar transceiver is small, lightweight, and it provides quick and precise measurements at a reasonable cost, mostly in this high-frequency band. The tests were carried out in free space on various polar fluid materials in a polypropylene test tube for determining signal sample delay and peak-to-peak amplitude of the received signals. The transmitting and receiving proposed fabricated antennas are facing at an angle of 180 degrees to each other with a test object set in the middle. The podal and antipodal antenna has a maximum realized gain of up to 11.3 dBi and 10.4 dBi, respectively. Both the antenna work in the ultrawideband region. UWB antennas enable the effective utilization of bandwidth at high data rates communication. Radar with UWB antennas is frequently employed in a variety of applications due to their numerous benefits such as low power consumption and costs. The Xethru radar transceiver work under the ultra-wideband frequency ranges from 5.3 GHz to 8.8 GHz. Hence, the proposed podal and antipodal antennas have a bandwidth ranging from 2.4 to 16 GHz and can be used as a transducer for the Xethru radar module. The paper is arranged as follows: Section 2 presents the design of the proposed antenna and its feeding structure, Section 3 consists of the parametric study of simulated and measurement results and discussion, and Section 4 discusses the operation of the antenna with experimental results on different test samples with the proposed analogy. Finally, Section 5 is the conclusion of the work.

## 2. Antenna Design

### 2.1. Antenna Structure

**Figure 1:**
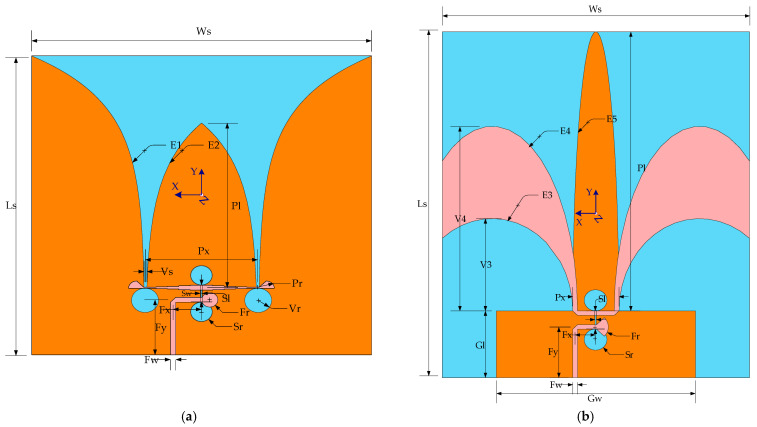
Structure of antenna with feed, substrate, and ground layer structure: (**a**) podal antenna; (**b**) antipodal antenna.

The configuration of the proposed double slot podal and antipodal Vivaldi antennas is shown in Figure 1a,b, respectively, with its optimal dimensions specified in Table 1.

**Figure 2:**
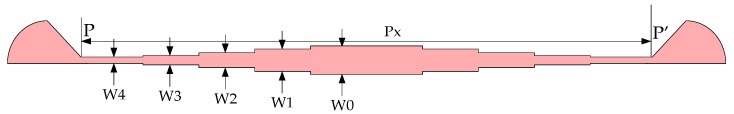
Structure of out-of-phase feeding microstrip line with Chebyshev multi-section 50 Ω to 100 Ω matching transformers designee using the following method [55]. The width of the calculated characteristics impedance of the three-section microstrip lines with reflection coefficient 0.05 for Taconic substrate of thickness 0.6 mm is W0 = 1.14 mm, W1 = 0.89 mm, W2 = 0.59 mm, W3 = 0.37 mm, and W4 = 0.25 mm. The length of each width of the section (W1 to W4) is a quarter of (Px/10) given that the total feeding length Px is 24 mm.

The feeding section of the antennas makes up the microstrip to slot line and slot to microstrip line power transitions shown in Figure 2, where both ends of the microstrip lines are out of phase with one another. Both antennas were designed on a Taconic substrate (εr = 4.5, tan δ = 0.0035). The size of the antenna is 70.69 mm × 72 mm × 0.6 mm and 89.88 mm × 74 mm × 0.6 mm. The top side of the podal antennas has a 50-ohm microstrip feedline to slotline and slotline to three-quarter wave Chebyshev transformer feeding microstrip line transition network whereas on the bottom side is a ground plane with two exponential tapering radiating patches. The antipodal antenna has an elliptical tapered patch variation through the edge of the ground plane. The T-junction, which is formed by connecting a slot line to a microstrip Chebyshev transformer feedline, splits the power to each line end by 180 ± 5 degrees, resulting in a steady radiation pattern. The three-section Chebyshev transformer matches a 50-ohm to 100-ohm microstrip line that feeds the podal antenna’s two exponential tapering slots E1 and E2. Similarly, in both the radiating and ground planes, the antipodal antenna includes three elliptical curves, E3, E4, and E5, which are stated in terms of the values of the parameters indicated in Table 1 by the following:

(1) $$\;E_{1}:x=\frac{1}{2}(P_{x}+V_{s}(exp(y\frac{ln\left(\frac{P_{x}}{V_{s}}\right)}{L_{s}-F_{y}-V_{r}})))\;;\;\;\;\;\;\;\;\;(0\leq y\leq \left(L_{s}-F_{y}-V_{r}\right)),$$

(2) $$\;E_{2}:x=\frac{1}{2}(P_{x}-V_{s}(exp(y\frac{ln\left(\frac{P_{x}}{V_{s}}\right)}{\frac{W4}{2}+P_{l}})))\;;\;\;\;\;\;\;\;\;\;\;\;\;\left(0\leq y\leq \frac{W4}{2}+P_{l}\right),$$

(3) $$\;E_{3}:y=\;\frac{2V_{3}}{G_{w}-P_{x}}\sqrt{{\left(\frac{G_{w}-P_{x}}{2}\right)}^{2}-x^{2}}\;\;;\;\;\;\;\;\;\;\;\;\;\;\;\;\;\;\;\;\left(-\left(\frac{W_{s}-G_{w}}{2}\right)\leq x\leq -\left(\frac{G_{w}-P_{x}}{2}\right)\right),$$

(4) $$\;E_{4}:y=\;\frac{2V_{4}}{G_{w}-P_{x}+2F_{w}}\sqrt{{\left(\frac{G_{w}-P_{x}+2F_{w}}{2}\right)}^{2}-x^{2}}\;;\;\;\left(-\left(\frac{W_{s}-G_{w}}{2}\right)\leq x\leq -\left(\frac{G_{w}-P_{x}+2F_{w}}{2}\right)\right),$$

(5) $$\;E_{5}:y=-\left(\frac{2(L_{s}-G_{l})}{P_{x}}\right)\;\sqrt{{\left(\frac{P_{x}}{2}\right)}^{2}-x^{2}}\;;\;\;\;\;\;\;\;\;\;\;\;\;\;\;\left(-\left(\frac{P_{x}}{2}\right)\leq x\leq 0\right),$$

### 2.2. Design of the Feeding Structure

The schematic of the proposed slot to microstrip T-junction power divider topology is shown in Figure 2, representing the feeding section of the fabricated podal antenna prototype. The three-quarter wave Chebyshev transformer feeding lines have a different width and are used to match the slot impedance of 100 ohms. In this figure, W1, W2, W3, and W4 represent the transition linewidth connecting the microstrip line and a radial stub.

**Figure 3:**
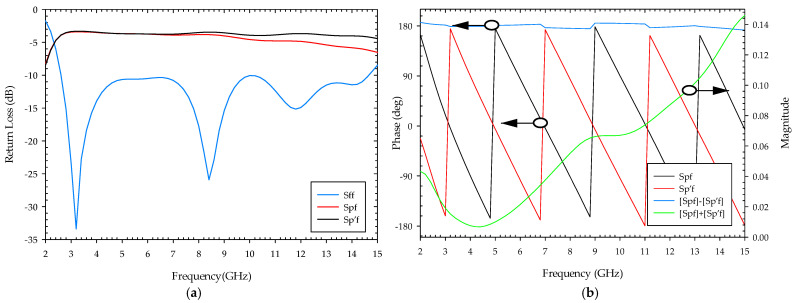
S-parameter with phase and magnitude at the end of the feeding network of the podal antenna: (**a**) feeding ends output return loss; (**b**) output port phases and a sum of magnitude.

If “p” and “p’” represent the power output at the two ends of the feeding network of the podal antenna, and “f” represents the input power through the SMA connector, the simulated S-parameter (Sff) of the power divider is below 10 dB, supporting the proposed antenna’s operating frequency range of 2.5–14.6 GHz, as shown in Figure 3a. The simulated insertion loss (Spf and Sp’f) spans from 4.4 dB to 6.5 dB. As shown in Figure 3b, the power divider yields equal power divisions with a phase difference (Spf − Sp’f) of 180 ± 5 degrees and almost equal and opposite magnitude between the two outputs.

## 3. Results and Discussion

**Figure 4:**
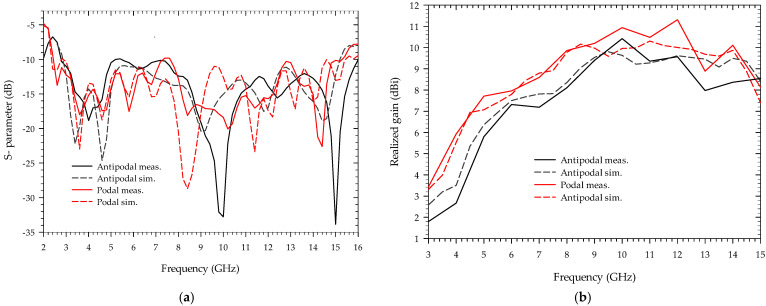
Simulated and measured result of the antenna: (**a**) return loss; (**b**) realized gain.

Using commercially available high-frequency structure simulator (HFSS) software, the proposed antenna is optimized and modeled. The simulation and measurement results are depicted in Figure 4. Figure 4a displays that the suggested podal and antipodal antennas have an impedance bandwidth of less than 10 dB between (2.4–15.4 GHz) and (2.8–16 GHz). Figure 4b shows that the realized gain is less than 11.3 dBi throughout the whole bandwidth, with a close agreement between measured and simulated outcomes. At 10, 12, and 13 GHz, the simulated and measured results differ slightly, which can be attributed to connector losses due to dimension imperfection and parasitic effect, fabrication errors during the etching process, and inadequate soldering of the feed line to the connector, and substrate properties.

**Figure 5:**
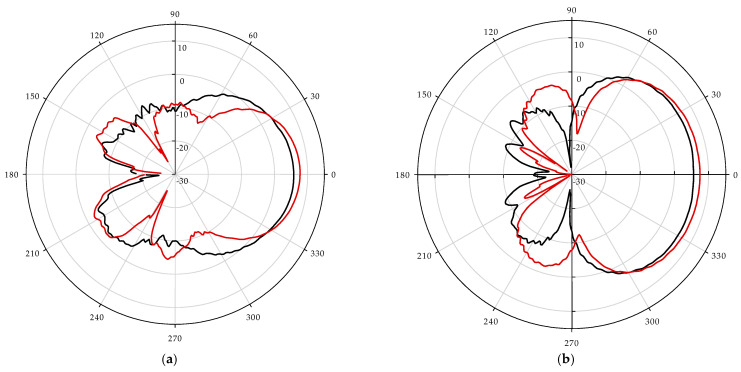

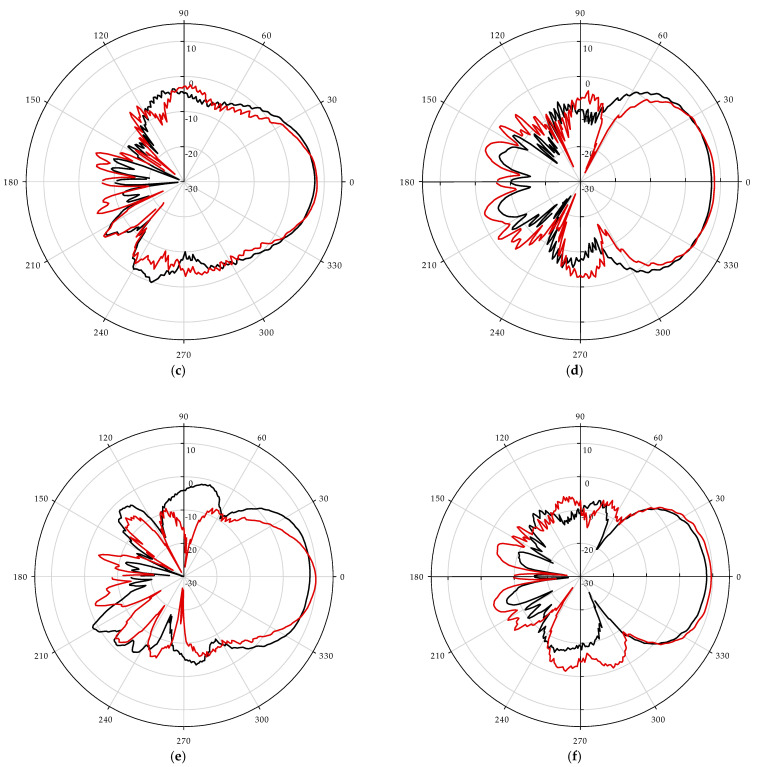

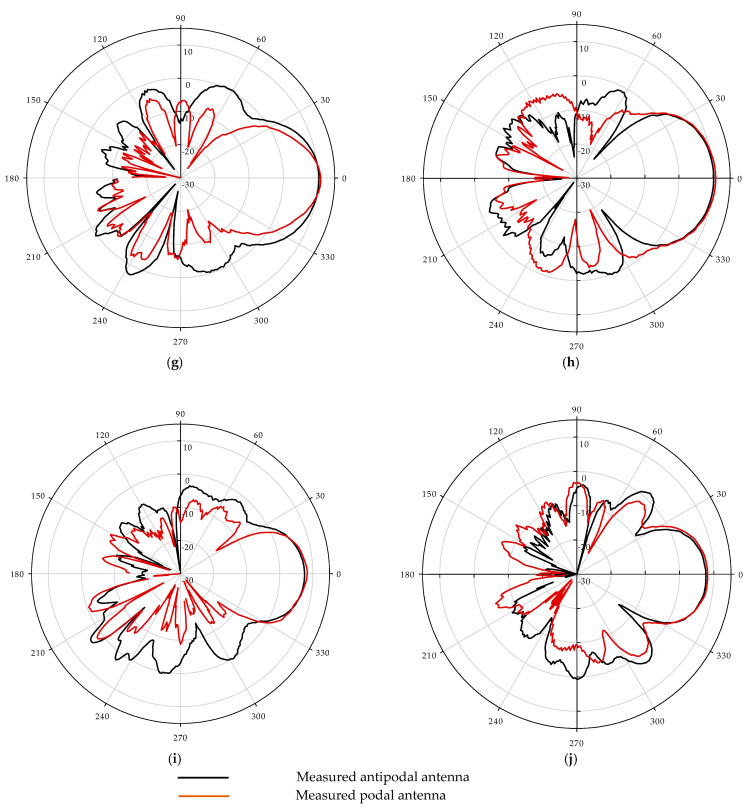
Measured far-field radiation pattern at E-plane (**a**,**c**,**e**,**g**,**i**) and H-plane (**b**,**d**,**f**,**h**,**j**) at frequency 5, 6, 8, 10, and 13 GHz respectively.

**Figure 6:**
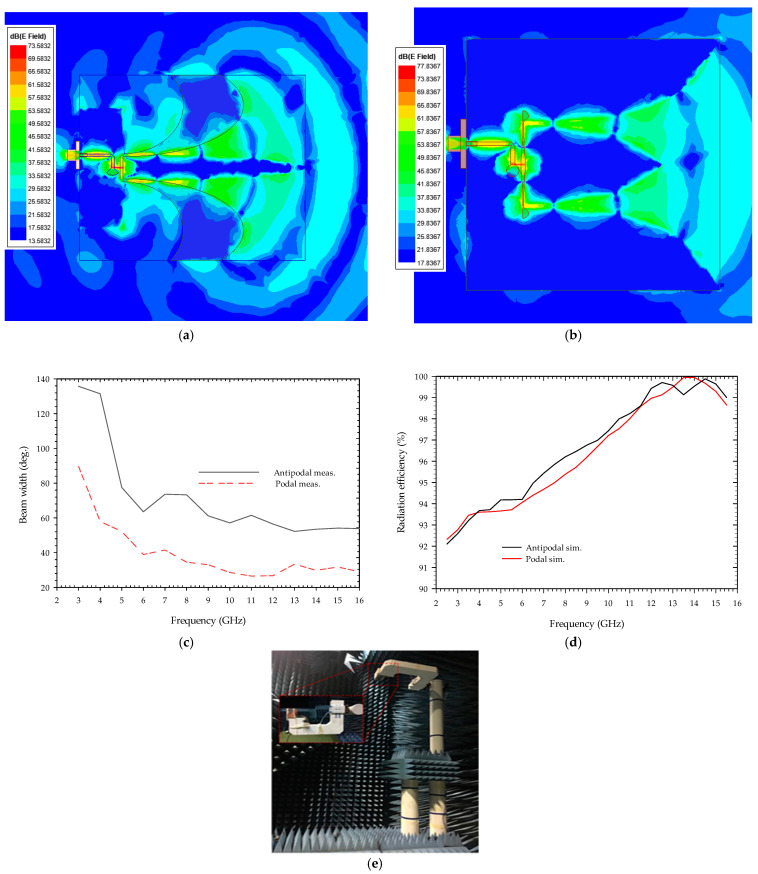
Simulated electric field distribution of the antenna and measured beamwidth: (**a**) 7 GHz antipodal antenna; (**b**) 7 Hz podal antenna; (**c**) Variation of beam width with frequency; (**d**) Simulated radiation efficiency of the antenna; (**e**) Measurement setup for the fabricated antennas.

Figure 5 illustrates the measured 2D radiation patterns of the designed antennas at 5, 6, 8, 10, and 13 GHz frequencies in the anechoic chamber room as shown in Figure 6c, using a far-field analysis system. The radiation patterns of the antenna are almost directional in both the E-plane (x–y plane) and the H-plane (z–y plane), which is one of the required directive properties for podal and antipodal antennas. Figure 6a,b show the simulated electric field distribution at 7 GHz; it can be seen that the electric field radiated due to a change in surface current at each tapering slot of the antennas superimposed to form a directive beam. Similarly, Figure 6c represents the measured 3 dB beamwidth of the constituent antenna whereas Figure 6d signifies simulated radiation efficiency.

The radiation performance and feed system of the suggested antennas are compared to that of previous known podal and antipodal antennas in Table 2. As indicated in the table, most antenna systems employ a T-junction power divider. In comparison to the existing ultra-wideband antennas, the suggested antenna offers feed management, compact size, and gain.

## 4. Experimental Study and Results

After testing the suggested antenna design, the experiment was carried out in a controlled environment for measuring the fluid’s properties based on Poiseuille’s law hypothesis. The test’s goal is to measure and evaluate the change in received signals transmitted through fluid samples placed in a polypropylene test tube diameter of 3 cm and 1 mm in thickness positioned within these two transmitting and receiving antennas in both podal and antipodal design configurations. These investigations are intended to build up measurement techniques of fluid resistance over transmitted waves for assessing fluid characteristics over different test parameters and identifying the materials being used through radar-based systems. As a material-under-test (MUT), 47 ml of seven different polar fluid samples are taken, namely ethanol, methanol, 2-propanol, acetonitrile, and distilled water.

### 4.1. Experimental Setup

**Figure 7:**
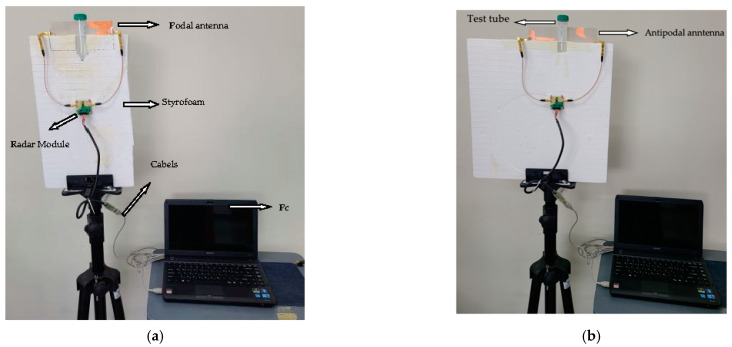

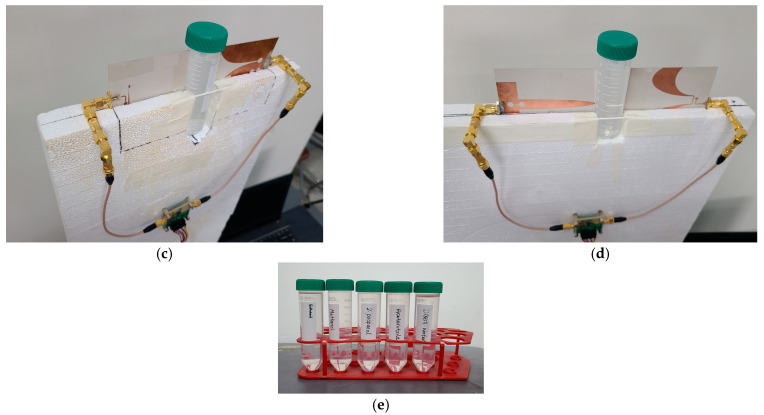
Experimental measurement setup: (**a**) proposed podal antenna scanning the MUT; (**b**) proposed antipodal antenna scanning MUT; (**c**) podal antenna with MUT side view; (**d**) antipodal antenna MUT side view and; (**e**) Test tubes containing the specimen.

As illustrated in Figure 7, the setup includes a UWB radar module (NVA-X2 R661 from Xethru Co., Oslo, Norway), a tripod stand, RF cables, supporting Styrofoam, a test tube containing the specimen, connectors connecting to the PC, and proposed antenna modules. To house the test specimen (Figure 7e), the Styrofoam is grooved in the shape of a test tube. The two antennas facing each other are mounted in the Styrofoam, with the specimen at the center as shown in Figure 7c,d.

**Figure 8:**
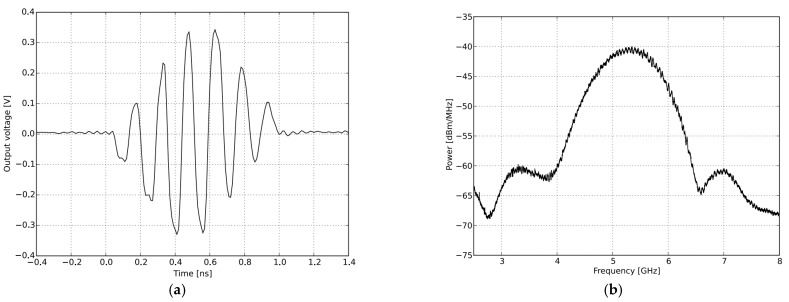
The Transmitted Pulse Shape and frequency spectrum of IR-UWB radar for PGSelect = 0: (**a**) transmitted signal impulse in the time domain; (**b**) transmitted signal impulse in the frequency domain.

The NVA-X2 R661’s chip generates and transmits UWB pulses of high-order Gaussian impulse signals with several GHz bandwidths and signal durations in the nanosecond range. The high-frequency signal was chosen based on the antenna’s maximum gain and return loss, as well as the radar’s capacity to transmit deep within the object with great resolution. The impulse signals were tuned around the antenna’s operational bandwidth, by a PGselect input with a peak-to-peak output amplitude of around 0.54 to 0.72 volts. The criterion of $\lambda_{c}/4$ satisfied the minimum separation of antennas distance, of 3 cm facing parallel to each other. Figure 8 illustrates the time and frequency domain responses in nanoseconds for the chosen ($f_{c}$) at 5.3 GHz.

### 4.2. Signal Analyzing

**Figure 9:**
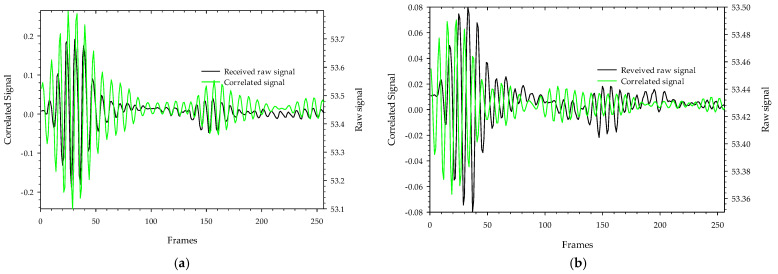
The received pulse shape and correlated signals from IR-UWB radar for 5.3 GHz transmission:(**a**) without MUT; (**b**) with MUT.

**Figure 10:**
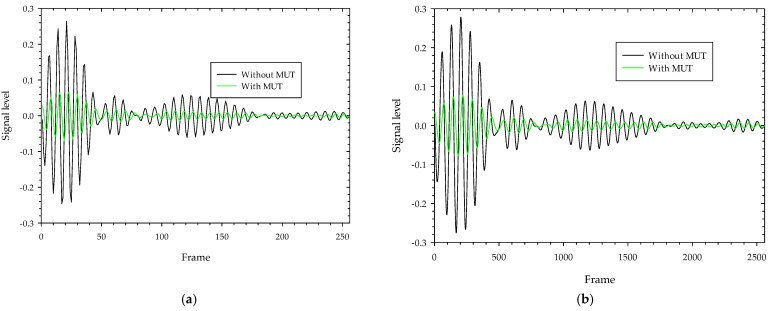
Correlated received pulse signals with and without MUT (ethanol) for PGSelect = 0; (**a**) correlated signals; (**b**) resample correlated signals.

**Figure 11:**
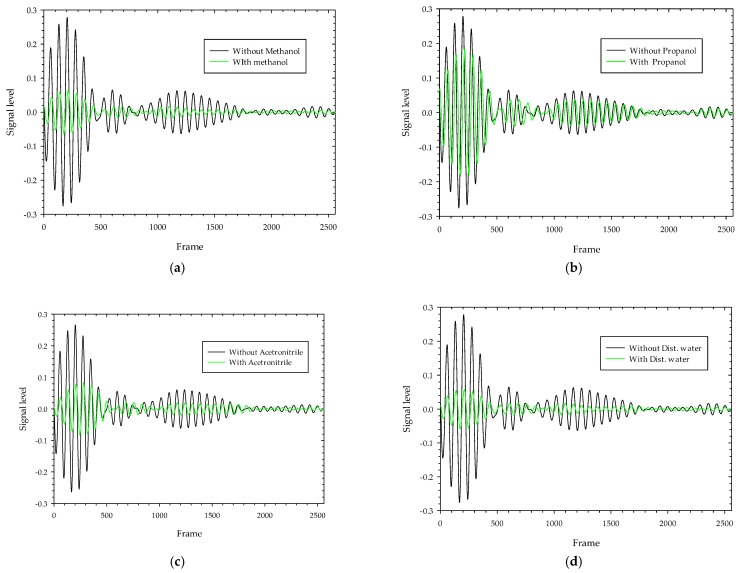
Resampled correlated received pulse signals with and without MUT at 5.3 GHz transmission using podal antenna: (**a**) Methanol; (**b**) Propanol; (**c**) Acetonitrile; (**d**) Distilled water.

Background signal and noise are inherent in the transmitted signals as they pass through the test specimen, so the received signals are significantly attenuated. The received raw signal is cross-correlated with the template signal (Figure 8a), which is generated by the X2 chipset to yield a correlated signal. This aids the signal in obtaining the maximum signal-to-noise ratio and correlates the received signal pattern with a low signal variation. The Xethru radar module’s PGSelect command is used to adjust the list of frequencies of the transmitted impulse signal. Figure 9a,b depict the raw signal and correlated signal with and without MUT on 5.3 GHz transmitted impulse signals where we can see that the level of received signals when passing through MUT are highly attenuated and delayed due to signal losses and absorption by the MUT. The correlated signals obtained with and without MUT (ethanol) as shown in Figure 10a are resampled 10:1 of the original rate (Figure 10b) to fill in all the missing points at the peaks and smooth the signals between the two frame positions, and finally, 150 samples around the maximum peak value of the signals obtained with and without MUT are chosen and then cross-correlated to detect the highest delay points. The maximum delay duration (Δ*t*) of the transmitted signal in a MUT sample is found by multiplying the radar sampling periods by the delay points. Figure 11 depicts the resampled signal levels at various MUT. As seen in the figures, each MUT corresponds to a different signal level and delay in a frame, which is one of the unique traits used to anticipate the MUT’s samples. Finally using these characteristics, the time delay and attenuation of transmitted electromagnetic waves at various radar transmission frequencies in a MUT are estimated on electromagnetic wave analogue based on Poiseuille’s law hypothesis and used to determine the fluid characteristic using Equation (10).

### 4.3. Working Assumption

The flow rate of liquid, *Q*, in a cylindrical tube is determined by Poiseuille’s law [60]

(6) $$Q=\frac{\left(P_{2}-P_{1}\right)\pi r^{4}}{8\xi l},$$

where $P_{2}$ and $P_{1}$ are the pressures at the tube’s input and exit ends. r and l are the tube’s inner diameter and length and ξ is the liquid viscosity. By rearranging the above equation, the viscosity can be calculated as

(7) $$\xi =\frac{\left(P_{2}-P_{1}\right)\pi r^{4}}{8Ql},$$

The relationship between flow rate, pressure difference, tube length and radius, and fluid viscosity is probably interpreted by developing an analogy between the laminar flow of fluid in a tube and the travel of electromagnetic waves through a material that has a resistive property to slow the wave.

The pressure exerted per unit area by the transmitting wave through a podal or antipodal antenna having a width (*Ws*) on a test tube holding MUT is influenced by the change in velocity for a given time interval. If *P_2_* and *P_1_* are the pressures experienced and *Va, Vm* are the transmitting wave velocities in a test tube without and with MUT respectively, then the pressure *P_2_* exerted on a test tube without MUT is zero, whereas the pressure *P_1_* experienced by MUT is the difference in wave velocity (*Va* and *Vm*) per maximum time of flight (*t_m_*) for given tube inner diameter (l) containing MUT. The change in pressure per unit area is then calculated as

(8) $$P_{2}-P_{1}=\frac{\left(V_{a}-V_{m}\right)}{t_{m}},$$

The flow rate *Q* can be defined as the velocity of a wave per time of flight through a medium and is proportional to the factor of peak-to-peak received signal voltage level (*Pa* and *Pm*) with MUT and without MUT and can be expressed as

(9) $$Q=\frac{V_{m}}{t_{m}}\times \alpha ,$$

where, $\alpha =\frac{P_{m}}{P_{a}}$.

Equation (8) can be rearranged based on the time of flight. By replacing the terms *Va* and *Vm* with $l∕t_{a}\;$ and $l∕t_{m}$. r with half an antenna width, and rearranging Equations (8) and (9) on Equation (7), we obtain the following:

(10) $$\xi =\frac{\left(t_{m}-t_{a}\right)\pi {\left(\frac{W_{s}}{2}\right)}^{4}}{8\alpha t_{a}l}$$

Here $\left(t_{m}-t_{a}\right)$ is the maximum delay duration (Δ*t*) which can be calculated by multiplying the radar sampling periods by the delay points. $t_{a}$ is the time taken by radar impulse to cover the inner diameter l of the test tube without MUT in a test tube.

### 4.4. Measurements

**Figure 12:**
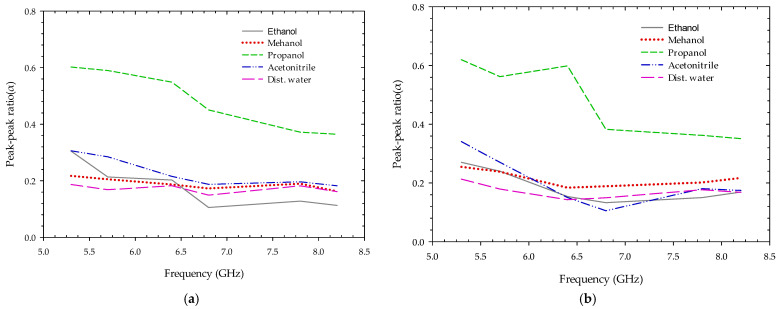
The ratio of peak-to-peak voltage level with and without fluid MUT at different transmitted impulse frequency transmission trough using (**a**) podal antenna; (**b**) antipodal antenna.

The experiments were performed in free space on a range of polar fluid materials in a 1 mm thick polypropylene test tube, including ethanol, methanol, propanol, Acetonitrile, and distilled water around 25 degrees Celsius in its pure form. The transmitting and receiving antennas are positioned at a 180-degree angle to each other, with a test item in the center. With a center frequency ($f_{c}$) of 5.3 GHz, 5.7 GHz, 6.4 GHz, 6.8 GHz, 7.8 GHz, and 8.2 GHz, and a peak-to-peak output amplitude of 0.54 to 0.72 volts, the transmitted impulse signals were adjusted around the antenna’s operational bandwidth. The resistive property of the MUT is calculated by knowing the values of various measured parameters for each transmitted frequency. As shown in Figure 12, the factor α for each fluid MUT at various transmitted radar impulse frequencies using both podal and antipodal antennae are distinct from each other.

**Figure 13:**
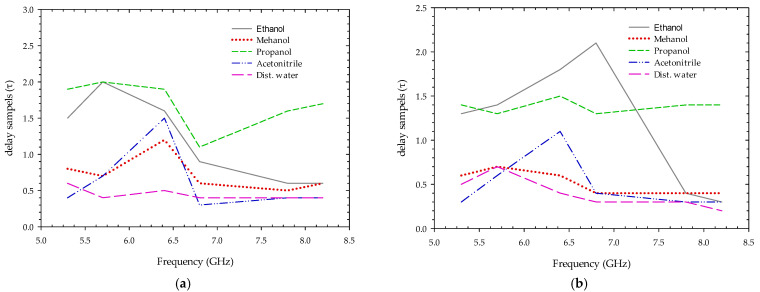
Delay in samples at different transmitting impulse frequencies trough MUT using (**a**) podal antenna; (**b**) antipodal antenna.

**Figure 14:**
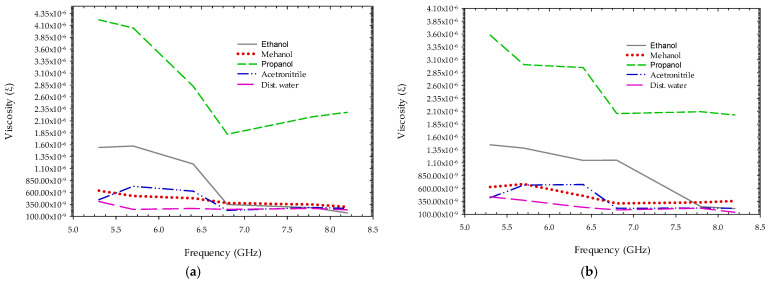
Resistivity experienced by a wave at different transmitting impulse frequencies through MUT using (**a**) podal antenna; (**b**) antipodal antenna.

Similarly, delay in a sample obtained from cross-correlation between the two-sample frames of MUT and without MUT are plotted in Figure 13. Because higher frequency signals attenuate faster than lower frequency signals, the signal-to-noise ratio is very low at higher frequencies, losses in the connecting cables, as well as parasitic effects on the soldered area around the SMA connector, affecting the measurement and delay of the samples. Figure 14 depict the resistivity or viscosity due to MUT on electromagnetic wave transmitted at different impulse frequencies using Equation (10). From the plot, we can see the electromagnetic wave interacting differently for each fluid sample, which may be used to forecast the nature of the fluid and its behavior for different transmission frequencies.

## 5. Conclusions

**Figure 15:**
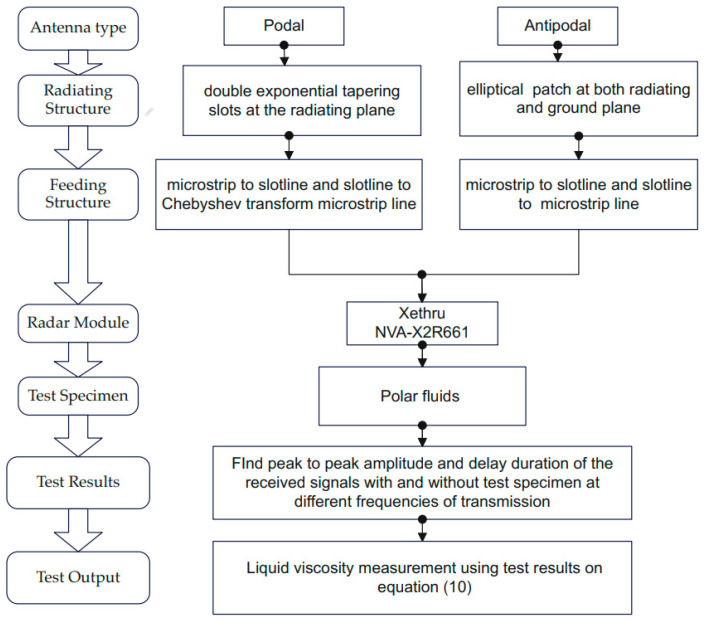
Graphical illustration of the proposed idea.

A feed system consisting of a high gain podal and antipodal antenna on a single substrate layer using a power splitter based out of phase uniplanar power divider approach has been presented. The antenna exhibits a uniform amplitude out of phase uniplanar power divider feeding methodology over a large frequency range. The feeding has a T-junction formed by a slot line and a microstrip line for both podal and antipodal antenna designs. The podal microstrip feed line consists of Chebyshev multi-section matching transformers. The proposed antennas have maximum realized gain up to 11.3 dBi and a 3 dB beamwidth range from 89.6 to 29.01 degrees for podal and 10.4 dBi, 135 to 39.5 degrees for antipodal antennas, respectively, within ultra-wideband regions of bandwidth from (2.4–15.4 GHz) and (2.8–16 GHz). Broad bandwidth, high gain, and strong directivity are all benefits of the suggested antenna, making it a viable option for applications that need broad bandwidth communication. The feeding system is compact, eliminates the beam-splitting effect, and significantly enhances the radiation directivity of the antenna arrays. The fabricated antenna is deployed in the detection of the MUT samples, and its viscous property is based on Poiseuille’s law hypothesis. To begin, radar scans were taken on the samples, and delay in the scanned signal from with and without MUT is calculated. Achieved delay is used to calculate the resistance or viscosity experienced by the flow of waves to the material. The MUT placed on the systems detecting the fluid nature and viscosity confirms that the proposed antenna is suitable for use in microwave liquid viscosity imaging and fluid prediction application. The overall graphical representation of a proposed idea in this paper can be illustrated in Figure 15.

## Figures and Tables

**Table 1:** Parametric dimensions of the proposed two antennas structures.

Parameters	Podal Antenna (mm)	Antipodal Antenna (mm)
Ls	70.69	89.88
Ws	72	74
Fw	1.14	1.14
Fy, Fx, Fr, Sr	13, 6.01, 1.71, 2.28	13, 6.0, 3.42, 2.73
Sl, Sw	3.9, 0.28	4.74, 0.3
Px	23.78	11.14
P1	39	72.56
Vr	2.85	--
V3	--	19.43
V4	--	20.57
Gl	--	17.31
Gw	--	48
Vs	0.58	--
Pr	1.71	--

**Table 2:** Comparison of the proposed antennas with other antennas in terms of gain, size, and feed system.

Ref.	Frequency Range (GHz)	Feed System	Size (mm^2^)	Gain (dBi)
[52]	8–12	SIW 1 × 8 power divider	100 × 57	12
[56]	2.5–15	T-junction power divider	120 × 80	14.5
[57]	26.1–28.3	SIW Right-Angled Power Dividers	--	8.5
[58]	7–32	microstrip feeding	140 × 66	12
[59]	25–31	4 × 4 butler matrix	29.52 × 20.52	10.2
Proposed antenna (podal)	2.4–15.4	T-junction power divider with slot and Chebyshev microstrip line	70.69 × 72	11.3
Proposed antenna (antipodal)	2.8–16	T-junction power divider with slot microstrip line	89.88 × 74	10.4

## Data Availability

The data presented in this study are available on request from the corresponding author.
